# Supplementary material for: Physically and Chemically Stable Anion Exchange Membranes with Hydrogen-Bond Induced Ion Conducting Channels
Source: Polymers (Basel). 2022 Nov 15;14(22):4920. doi: 10.3390/polym14224920 (PMC9696997; doi:10.3390/polym14224920)
Supplement: Supplementary file 1 [file polymers-14-04920-s001.zip › polymers-1958780-supplementary.pdf]

Supplementary materials

Physically and Chemically Stable Anion Exchange Membranes with Hydrogen-bond Induced Ion Conducting Channels

Chengpeng Wei, Weisheng Yu, Liang Wu, Xiaolin Ge * and Tongwen Xu *

Anhui Engineering Laboratory of Functional Membrane Materials and Technology, Collaborative Innovation Centre of Chemistry for Energy Materials, School of Chemistry and Material Science, University of Science and Technology of China, Hefei 230026, China

* Correspondence: gexl@ustc.edu.cn (X.G.); twxu@ustc.edu.cn (T.X.)

1. Experimental details

1.1. Ion exchange capacity (IEC) determination

The theoretical IECs of the CPBTT-x AEMs were calculated based on feeding ratio, while the experimental IECs were determined by Mohr titration. Typically, the membrane sample was first immersing in 1 M NaCl aqueous solution for 24 h. Then the sample was washed with DI water thoroughly and dried completely under vacuum, and the dry mass of the membrane was recorded as W_{dry}. After that, the membrane was immersed in 0.5 M Na_2SO_4 aqueous solution for 24 h to completely exchange the Cl^- with SO_4^{2-}. The solution was titrated with the 0.01 M $AgNO_3$ standard solution using K_2CrO_4 as an indicator, and the volume of the consumed $AgNO_3$ solution was recorded as V_{AgNO3}. The IECs of the AEMs were calculated according to the following formula:

$$IEC(mmol\ g^{-1}) = \frac{0.01 \times V_{AgNO_3}}{W_{dry}}$$

1.2. Water uptake (WU) and swelling ratio (SR) measurement

The WU and SR of the AEMs were measured according to the change in membrane weight and liner dimension between the wet and dry state. The membrane sample was immersed in 1 M NaOH aqueous solution for 24 h to exchange the accompanied ion to OH^- form. After washing the membrane completely with DI water, the membrane was soaking in DI water at different temperature for 6 h to achieve the adsorption equilibrium. After completely

removing the excessive surface water, the weight and length of the wet membrane were measured immediately and recorded as W_{wet} *and* L_{wet} respectively. Then, the membrane was dried under vacuum at 80 °C for 24 h, and the weight (W_{dry}) and length (L_{dry}) of the dry membrane were measured. The WU and SR were calculated according to the following formulas:

$$WU\ (\%) = \frac{W_{wet} - W_{dry}}{W_{dry}} \times 100\%$$

$$SR\ (\%) = \frac{L_{wet} - L_{dry}}{L_{dry}} \times 100\%$$

1.3. Hydroxide conductivity measurement

The in-plane OH^- conductivities of the AEMs were measured using a four electrode AC impedance technique (Auto lab Zahner Zennium E, Germany.). The wet membrane sample (4 cm × 1 cm, with thickness of 50 um) in OH^- form was placed into the Teflon cell equipped with two outer current-carrying electrodes and two inner potential-sensing electrodes, and then the cell was immersed in DI water immediately. The impedance spectroscopy was recorded at given temperature under galvanostatic mode with the frequency range of 1 M Hz to 10 Hz and an AC current amplitude of 10 uA. The OH^- conductivity (σ, mS cm^{-1}) was calculated according to the following formula:

$$\sigma = \frac{L}{RWd}$$

where *R*, *L*, *W* and *d* represent the impedance, distance between the two potential sensing electrodes (herein 1 cm), width and thickness of the membrane sample, respectively.

The activation energy for OH^- conduction of the AEMs was calculated based

on the following formula:

$$\ln(\sigma) = \ln A - \frac{E_a}{R}\left(\frac{1}{T}\right)$$

Where A, R represent the Arrhenius constant and universal gas constant respectively, T represents the temperature, and E_a represents the activation energy.

1.4.Alkaline stability measurement

The alkaline stabilities of the AEMs were evaluated by immersing the membranes in 1 M NaOH at 80 ^{o}C for 1080 h. The IEC values of the AEMs during the alkaline treatment were monitored.

1.5. Single cell performance

The membrane electrode assembly for single cell performance was fabricated according to our previous work [60]. The poly(fluorene-co-biphenyl N,N′-dimethylpiperidinium) (PFBP-14) copolymer reported by Lee's group was used as the ionomer [61]. The H_2/O_2 single cell performance of the AEMs was analyzed by a 890e multi-range fuel cell test station. PtRu/C and Pt/C (both 60 wt% metal content) were used as the catalysts for anode and athode, respectively. The ionomer was firstly dissolved in DMSO to prepare a 5 wt% polymer solution, then the solution was added to the suspension of catalyst in isopropyl alcohol/DI water (4:1) to prepare the catalyst ink (20 wt% ionomer and 80 wt% catalyst). The obtained ink was sonicated for 1 h and sprayed onto a carbon paper (Toray TGP-H-060) to fabricate the gas diffusion electrodes

(GDEs). The catalyst loading was controlled at 0.5 mg cm^{-2} and the electrode area was 5 cm^2. The fabricated GDEs and AEM were immersed in 1 M NaOH for 12 h to convert to OH^- form, and then they were washed with DI water. Finally, the AEM was sandwiched between the two GDEs to obtain the membrane electrode assembly. The H_2/O_2 single cell performance of the AEMs was analyzed by a 890e multi-range fuel cell test station in a galvanic mode at 70 °C with a 1000 mL min^{-1} flow rate (for both H_2 and O_2, 100% RH, without back pressure).

2. General properties of the AEMs

Table S1. IEC, WU, SR and OH^- conductivity of the AEMs

sample	IEC (mmol g^{-1})		WU（%）		SR（%）		σ（OH^-）	
	Theo[a]	exp[b]	30 °C	90 °C	30 °C	90 °C	30 °C	90 °C
CPBTT-0.4	1.70	1.48	27.9	32.5	8.8	9.8	39.9	97.2
CPBTT-0.5	1.97	1.63	49.7	63.1	11.8	14.5	47.9	111.2
CPBTT-0.6	2.20	1.77	80.2	99.3	17.6	20.3	59.1	122.9

[a] Theoretical IECs calculated by feeding ratio. [b] experimental IECs determined by Mohr titration.

3. SEM images of the AEMs.

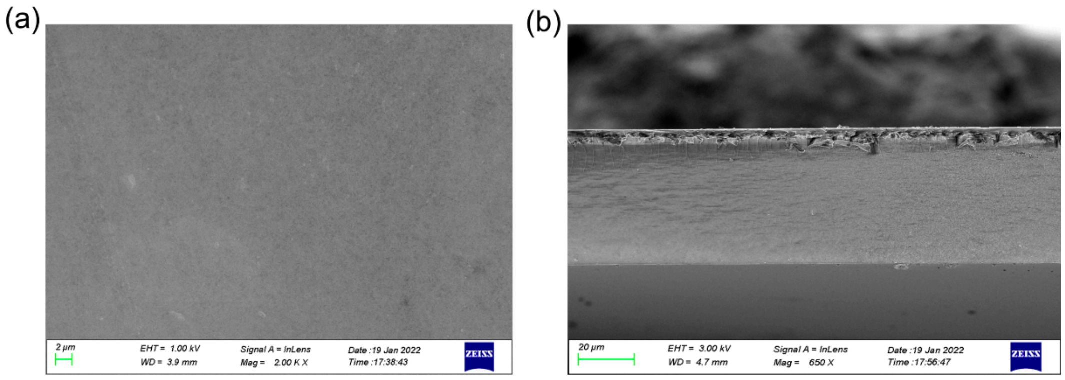

Figure S1. (a) Surface and (b) cross-section SEM images of the CPBTT-0.6

membrane.

4. Differential thermal gravity (DTG) curves of the AEMs.

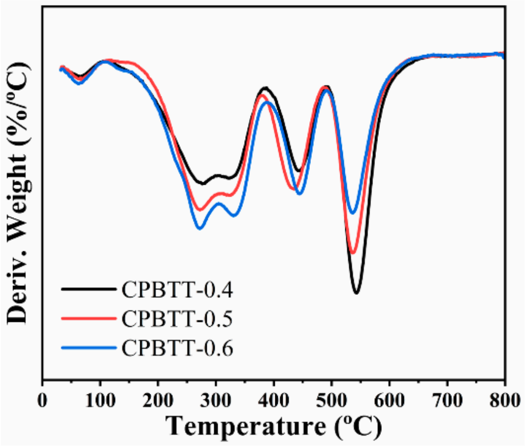

Figure S2. DTG curves of the AEMs of the CPBTT-x AEMs.

5. Alkaline stability of the AEMs.

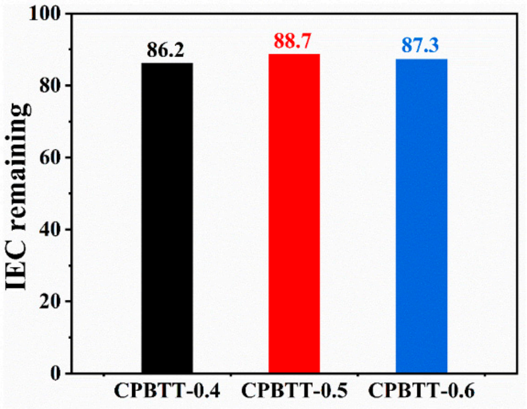

Figure S3. IEC remaining of the CPBTT-x AEMs after durability testing (1 M NaOH at 80 °C for 1080 h).

6. Comparison of the AEMFCs performance of the CPBTT-0.6 AEM with other reported AEMs.

Table S2. Comparison of the AEMFCs performance of the CPBTT-0.6 AEM with other reported AEMs

samples	σOH^- at 30 °C (mS cm^{-1})	Testing temperature (°C)	H_2/O_2 flow rate (mL min^{-1})	Backpressure (MPa) anode/cathode	peak power density (mW cm^{-2})	Reference
CPBTT-0.6	59.1	70	1000/1000	0/0	555	This work

PDTP-20	50	60	400/400	0/0	522	[57]
QPEPFpi-70	44	80	200/200	0.1/0.1	506	[62]
PDTP-25	80	80	1000/1000	1.3/1.3	2580	[17]
PDTP	61	80	1000/1000	0/0	1812	[63]
PQP-100	67	60	500/500	0/0	496	[64]
x-PFTP-PS-10	65	80	1000/1000	1.3/1.3	1610	[65]
PAP-TP-85	88	95	600/600	0.1/0.1	860	[1]
O-PDQA-3	61	70	1000/1000	0/0	1180	[39]
x-PFTP-10	68	80	1000/1000	0/0	1610	[66]
QPCTP-10	75	80	1300/1300	0.02/0.02	1720	[21]

References

1. Wang, J.; Zhao, Y.; Setzler, B.P.; Rojas-Carbonell, S.; Ben Yehuda, C.; Amel, A.; Page, M.; Wang, L.; Hu, K.; Shi, L.; et al. Poly(aryl piperidinium) membranes and ionomers for hydroxide exchange membrane fuel cells. Nat. Energy 2019, 4, 392–398.

17. Chen, N.; Hu, C.; Wang, H.H.; Kim, S.P.; Kim, H.M.; Lee, W.H.; Bae, J.Y.; Park, J.H.; Lee, Y.M. Poly(Alkyl-Terphenyl Piperi-dinium) Ionomers and Membranes with an Outstanding Alkaline-Membrane Fuel-Cell Performance of 2.58 W cm−2. Angew. Chem. Int. Ed. 2021, 60, 7710–7718.

21. Yuan, W.; Zeng, L.; Jiang, S.; Yuan, C.; He, Q.; Wang, J.; Liao, Q.; Wei, Z. High performance poly(carbazolyl aryl piperidinium) anion exchange membranes for alkaline fuel cells. J. Membr. Sci. 2022, 657, 120676.

39. Zhang, J.; Zhang, K.; Liang, X.; Yu, W.; Ge, X.; Shehzad, M.A.; Ge, Z.; Yang, Z.; Wu, L.; Xu, T. Self-aggregating cationic-chains enable alkaline stable ion-conducting channels for anion-exchange membrane fuel cells. J. Mater. Chem. A 2021, 9, 327–337.

57. Wang, X.; Qiao, X.; Liu, S.; Liu, L.; Li, N. Poly(terphenyl piperidinium) containing hydrophilic crown ether units in main chains as anion exchange membranes for alkaline fuel cells and water electrolysers. J. Membr. Sci. 2022, 653, 120558.

60. Liang, X.; Shehzad, M.A.; Zhu, Y.; Wang, L.; Ge, X.; Zhang, J.; Yang, Z.; Wu, L.; Varcoe, J.R.; Xu, T. Ionomer Cross-Linking Immobilization of Catalyst Nanoparticles for High Performance Alkaline Membrane Fuel Cells. Chem. Mater. 2019, 31, 7812-7820.

61. Chen, N.; Wang, H.H.; Kim, S.P.; Kim, H.M.; Lee, W.H.; Hu, C.; Bae, J.Y.; Sim, E.S.; Chung,

Y.-C.; Jang, J.-H.; et al. Poly(fluorenyl aryl piperidinium) membranes and ionomers for anion exchange membrane fuel cells. Nat. Commun. 2021, 12, 2367.

62. Liu, J.; Gao, L.; Di, M.; Hu, L.; Sun, X.; Wu, X.; Jiang, X.; Dai, Y.; Yan, X.; He, G. Low boiling point solvent-soluble, highly conductive and stable poly (ether phenylene piperidinium) anion exchange membrane. J. Membr. Sci. 2022, 644, 120185.

63. Hu, C.; Park, J.H.; Kim, H.M.; Wang, H.H.; Bae, J.Y.; Liu, M.-L.; Kang, N.Y.; Yoon, K.-s.; Park, C.-d.; Chen, N.; et al. Robust and durable poly(aryl-co-aryl piperidinium) reinforced membranes for alkaline membrane fuel cells. J. Mater. Chem. A 2022, 10, 6587-6595.

64. Liu, M.; Hu, X.; Hu, B.; Liu, L.; Li, N. Soluble poly(aryl piperidinium) with extended aromatic segments as anion exchange membranes for alkaline fuel cells and water electrolysis. J. Membr. Sci. 2022, 642, 119966.

65. Chen, N.; Hu, C.; Wang, H.H.; Park, J.H.; Kim, H.M.; Lee, Y.M. Chemically & physically stable crosslinked poly(aryl-co-aryl piperidinium)s for anion exchange membrane fuel cells. J. Membr. Sci. 2021, 638, 119685.

66. Chen, N.; Park, J.H.; Hu, C.; Wang, H.H.; Kim, H.M.; Kang, N.Y.; Lee, Y.M. Di-piperidinium-crosslinked poly(fluorenyl-co-terphenyl piperidinium)s for high-performance alkaline exchange membrane fuel cells. J. Mater. Chem. A 2022, 10, 3678-3687.
